# Over 100 Million Frames per Second 368 Frames Global Shutter Burst CMOS Image Sensor with Pixel-wise Trench Capacitor Memory Array †

**DOI:** 10.3390/s20041086

**Published:** 2020-02-17

**Authors:** Manabu Suzuki, Yuki Sugama, Rihito Kuroda, Shigetoshi Sugawa

**Affiliations:** Graduate School of Engineering, Tohoku University, 6-6-11-811, Aza-Aoba, Aramaki, Aoba-ku, Sendai, Miyagi 980-8579, Japan; yuki.sugama.r3@dc.tohoku.ac.jp (Y.S.); rihito.kuroda.e3@tohoku.ac.jp (R.K.); shigetoshi.sugawa.d4@tohoku.ac.jp (S.S.)

**Keywords:** burst CMOS image sensor, 3D stacking, analog memory, burst correlated double sampling

## Abstract

In this paper, a prototype ultra-high speed global shutter complementary metal-oxide-semiconductor (CMOS) image sensor with pixel-wise trench capacitor memory array achieving over 100 million frames per second (fps) with up to 368 record length by burst correlated double sampling (CDS) operation is presented. Over 100 Mfps high frame rate is obtained by reduction of pixel output load by the pixel-wise memory array architecture and introduction of the burst CDS operation which minimizes the pixel driving pulse transitions. Long record length is realized by high density analog memory integration with Si trench capacitors. A maximum 125 Mfps frame rate with up to 368 record length video capturing was confirmed under room temperature without any cooling system. The photoelectric conversion characteristics of the burst CDS operation were measured and compared with those of the conventional CDS operation.

## 1. Introduction

Ultra-high speed (UHS) imaging technologies with over 100 million frames per second (fps) high frame rate and long record length are desired for the elucidation of UHS phenomena. Visualization of UHS phenomena such as shockwave, microbubble, breakdown, plasma, and discharge requires a frame rate exceeding several million fps. It is expected that these UHS phenomena can be visualized in more detail by improve the performance of the UHS imaging system. Multi-framing cameras or streak cameras have been utilized in UHS imaging over 100 Mfps [1]. However, these imaging systems are relatively large-scale, have issues in portability and convenience, and have not become a tool for easily visualizing UHS phenomena in various scientific research and development fields. Therefore, UHS image sensors achieving over 100 Mfps is highly desired for a simpler camera system. Consequently, burst image sensors with on chip multiple frame memory have been actively researched. They include charge domain storage type having multiple photoelectrons storage node connected to photodiode (PD) such as multi-collection gate (MCG) [2,3] or lateral electric field modulator (LEFM) [4,5]. Very high time resolution such as less than 1 ns has been obtained by this scheme because that is determined by the photoelectrons transit time and the switching time of the charge storage node, respectively. However, the number of record length is limited to a few by this scheme. The burst complementary metal-oxide-semiconductor (CMOS) image sensor technology having multiple analog voltage domain memories connected to each pixel is another candidate to achieve such high frame rate with long record length and acceptable power consumption.

**Figure 1:**
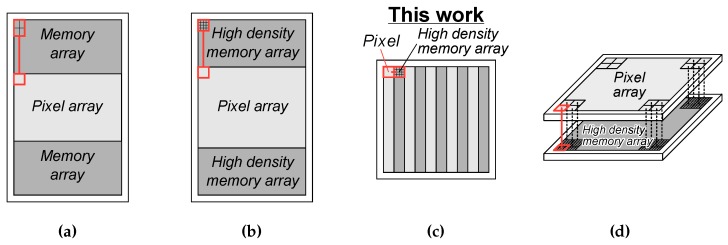
Schematic illustration of structures of burst ultra-high speed (UHS) complementary metal-oxide-semiconductor (CMOS) image sensor with on-chip memories; (**a**) the previously developed chip, (**b**) an improved structure of (**a**) with high density memory array, (**c**) a planar structure with pixel-wise high density memory array, and (**d**) a backside illuminated 3D stacked structure with pixel-wise interconnections.

Figure 1 shows structures of burst CMOS image sensors [6], where Figure 1a shows the previously developed chip with memory array placed on the periphery of pixel array achieved 20 Mfps with up to 256 record length [7,8], Figure 1b shows an improved structure of Figure 1a with increased number of pixels and record length by high density memory array [9,10], and Figure 1c shows a planar structure with pixel-wise high density memory array [11,12,13]. Recently, we have developed a prototype CMOS image sensor of Figure 1c with conventional planar MOS capacitor memory array [12,13]. It is mimicking a 3D stacked structure with pixel-wise interconnections for analog memory array placed beneath each pixel [14] shown in Figure 1d. It achieved over 100 Mfps with up to 80 frame video capturing by introducing the burst correlated double sampling (CDS) technique [13] which can minimize the frame period.

A high density memory integration is the key technology to achieve all the structures in Figure 1b–d with a sufficient number of record length. The thermal noise arising at the memory capacitors is one of the dominant noise sources in this type of image sensor, thus a relatively large capacitance value is required. Low leakage current and high uniformity are also required in order to maintain signal integrity. In order to meet such requirements, we have developed Si trench capacitor with improved effective capacitance density [9,10]. It achieved the capacitance density of about 30 fF/μm^2^. The developed prototype image sensor with the architecture shown in Figure 1b realized 10 Mfps UHS imaging with 960 record length with checkered pattern half pixel mode [10].

In the paper of IISW 2019 [15], we presented a burst CMOS image sensor with pixel-wise trench capacitor memory array to realize extremely high frame rate with longer record length. Over 100 Mfps with 368 frames imaging by using the developed burst CMOS image sensor has been demonstrated. In this paper, we additionally describe the detailed circuit design and the burst CDS operation we have proposed. The photoelectric conversion characteristics of the burst CDS operation are also reported with the comparison to the conventional CDS operation.

## 2. Design and Structure of Developed CMOS Image Sensor

**Figure 2:**
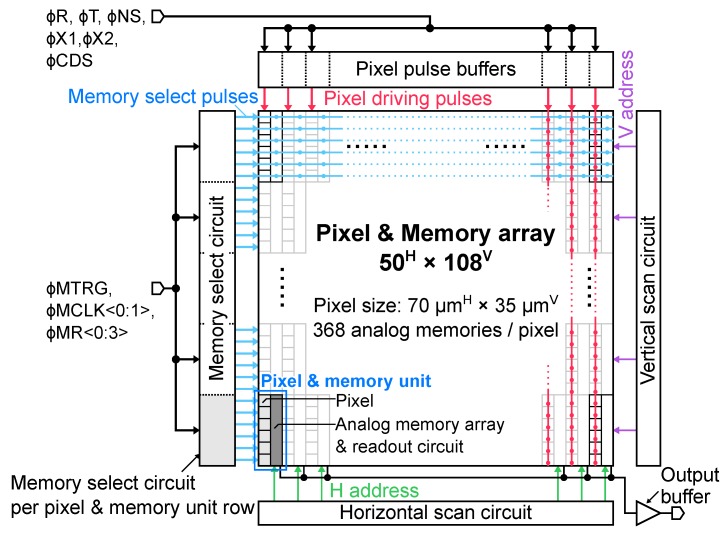
The block diagram of developed burst CMOS image sensor with in-pixel trench capacitor memory array.

Figure 2 shows the block diagram of the developed burst CMOS image sensor. The prototype chip has $50^{H}\times 108^{V}$ pixels with pixel-wise 368 trench capacitor memory array placed adjacent to each pixel. The pixel size is $35^{H}\;\mu m\times 35^{V}\;\mu m$ and the pixel pitch is $70^{H}\;\mu m\times 35^{V}\;\mu m$. Since this sensor is a prototype toward the pixel-wise 3D stacking type shown in Figure 1d, the pitches of the pixel and the pixel-wise analog memory are designed to be equal. Therefore, 3D stacked equivalent pixel pitch of the developed image sensor is 35 μm. The pixel and memory unit consists of six pixels and pixel-wise memory array and memory readout circuit for each pixel. The burst UHS video capturing is carried out by global shutter. The pixel pulse buffers driving all the pixels of each column are arranged in each pixel column, and memory select circuits for selecting each frame memory are arranged in each unit row. The pixel driving pulses (ϕR, ϕT, ϕNS, ϕX1, ϕX2, ϕCDS) are distributed to each pixel pulse buffer circuit, and the memory select control pulses (ϕMTRG, ϕMCLK<0:1>, ϕMR<0:3>) are distributed to each memory select circuit. A vertical and horizontal scan circuit and an analog output buffer are arranged to read out signals stored in the analog memories after the burst video capturing.

**Figure 3:**
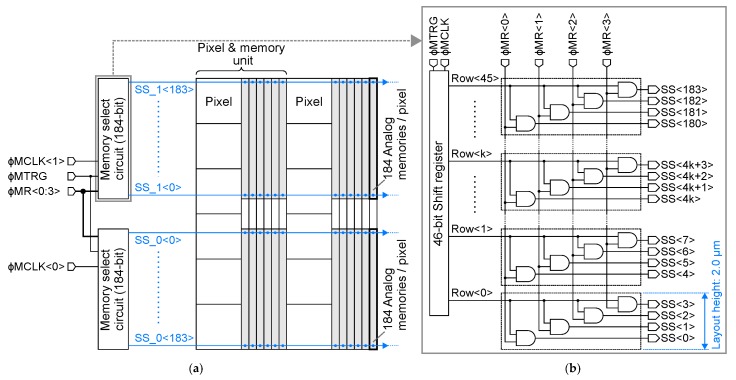
(**a**) The layout diagram of the memory select circuit for each pixel and memory unit row, (**b**) The circuit schematic diagram of the memory select circuit for 184 analog memories.

Figure 3a depicts the layout diagram of the memory select circuit for each unit row. The pixel-wise memory array is divided into upper and bottom parts in the unit, and the memory select circuit is also divided into two blocks in the same manner. Figure 3b shows the circuit diagram of the memory select circuit for 184 analog memories. The memory select circuit is composed of a combination of a 46-bit shift register and 4-bit demultiplexers connected to each output of the shift register. Where ϕMTRG is the trigger pulse for the shift register, ϕMCLK<0:1> are the clock pulses for the shift register, ϕRow<0:45> are the outputs of the shift register, and ϕMR<0:3> are demultiplexer select pulses. Each output of the shift register is branched into four signals by a demultiplexer. With this configuration, 184 analog memories for each pixel can be selected individually. This configuration was adopted, because the metal wires are efficiently routed under the constraint of the process design rule and the high flexibility of the memory selection.

**Figure 4:**
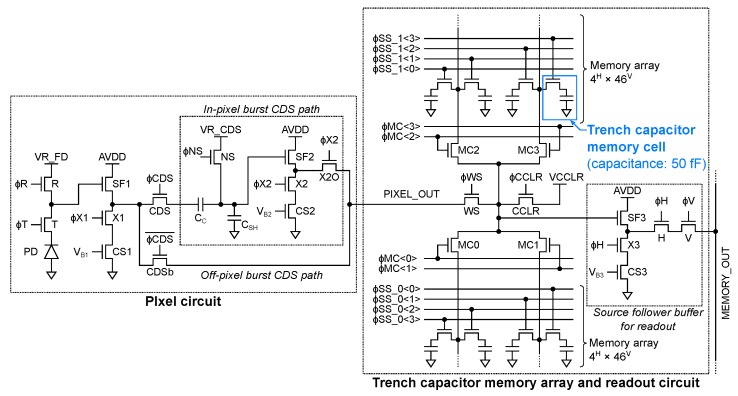
The circuit schematic of the pixel, the pixel-wise analog memory array, and the readout circuit of the developed burst UHS CMOS image sensor.

**Figure 5:**
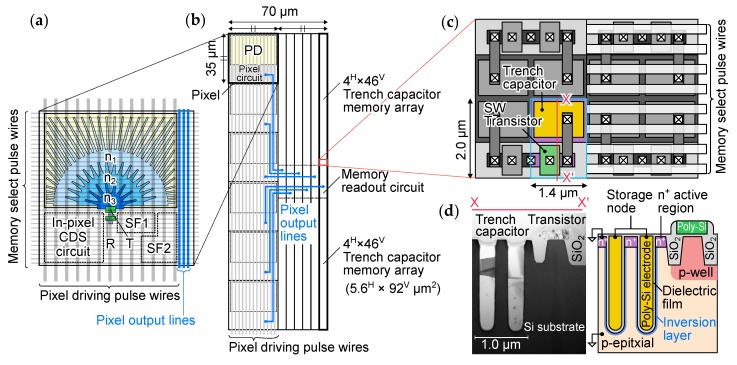
(**a**) Layout diagram of pixel, (**b**) arrangement of the pixel and memory unit for the developed image sensor, (**c**) layout diagram, and (**d**) cross sectional diagram of the trench capacitor memory.

Figure 4 shows the circuit schematic of the pixel, the pixel-wise analog memory array, and the readout circuit of the developed burst UHS CMOS image sensor. The layout diagram of the pixel is shown in Figure 5a. The pixel consists of a 30.00 μm × 21.34 μm high speed charge collection pinned photodiode (PD) using dopant concentration gradient and fringe electric field [16], a transfer switch (T), a floating diffusion (FD), a reset switch (R), a source follower (SF1), a select switch (X1), a current source (CS1), CDS bypass/select switches, and a CDS circuit with a SF buffer (SF2). In-pixel and off-pixel CDS operations are selectively available by using or bypassing the in-pixel CDS circuit. The pixel-wise trench capacitor memory array consists of $4^{H}\times 92^{V}$ one transistor one capacitor high density trench capacitor memory cells with capacitance of about 50 fF. The array is arranged at the center of the readout circuit and is divided into upper and bottom parts. The readout circuit consists of four column select switches (MC), a column reset switch (CCLR), a write select switch (WS), a SF buffer (SF3), a current source for SF3 (CS3), and select switches (X3, H, V). The horizontal and vertical addressing signal ϕH and ϕV are generated by the horizontal and vertical scan circuit shown in Figure 2. The WS switch is employed to equalize the input node capacitance of SF3 of each pixel during memory signal readout in order to equalize the charge division gain. When considering the 3D stacking of the pixel die and the memory array die, they can be divided at the node PIXEL_OUT.

Like other high speed imaging devices such as single photon avalanche diode (SPAD) array and time of flight image sensor, skew and jitter of shutter pulses supplied from outside the pixel array are challenges associated with speed improving and increasing pixel number. In the conventional CDS operation, the transfer pulse ϕT determines the shutter timing, and in the burst CDS operation, the memory selection pulse determines the shutter timing. Both pulses are supplied to the entire array from a buffer disposed closest to the array. The pixel pulse drives a buffer arranged for each pixel row and one buffer drives $108^{V}$ pixels. The memory selection pulse selects $50\times 6^{H}$ memories from the buffer of each pixel unit row. The skew is designed to be sufficiently small for the frame period. The jitter depends on the clock accuracy since the driving pulse is supplied from an external circuit. No special circuit for correcting jitter is provided inside the sensor. Further, although there is a possibility that jitter occurs in the internal circuit due to variation in threshold voltage of the sensor internal logic circuit, it is considered that the value is sufficiently small with respect to the frame period.

Figure 5b depicts the pixel and memory unit arrangement. One unit consists of six pixels, a memory array and a memory readout circuit for each pixel. The memory array has a very high aspect ratio, the vertical side being longer, because the memory select wires need to be routed horizontally as shown in Figure 5a,b. The number of pixels per unit was decided by balancing the record length and the load of the pixel output from the constraints of analog memory size, pixel pitch, and pixel output wiring length. The pixel driving pulse wires run vertically and the length of each pixel output wiring is made equal in order to equalize the load of the pixel output lines. Figure 5c,d shows the layout and cross-sectional diagrams of trench capacitor memory. Si trench capacitor with high integrity SiO_2_ dielectric film was employed for analog memory to obtain high capacitance with low leakage current and high uniformity [9,10]. The size of one transistor and one trench capacitor memory cell is $1.4^{H}\mu m\times 2.0^{V}\mu m$. It achieves about four times higher memory cell density than conventional planar MOS capacitor cell both using 0.18 μm CMOS technology [7,8].

**Figure 6:**
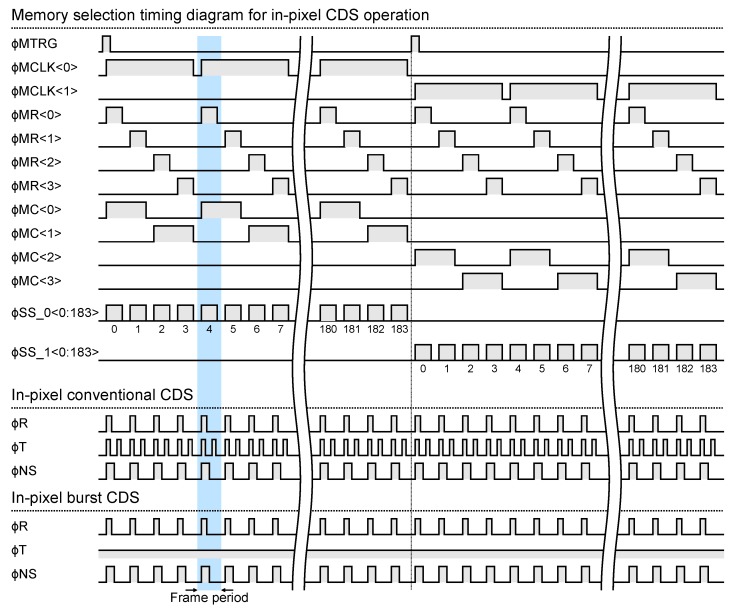
Timing diagram of the memory selection pulses and the pixel driving pulses at burst video capturing in-pixel correlated double sampling (CDS) operation.

**Figure 7:**
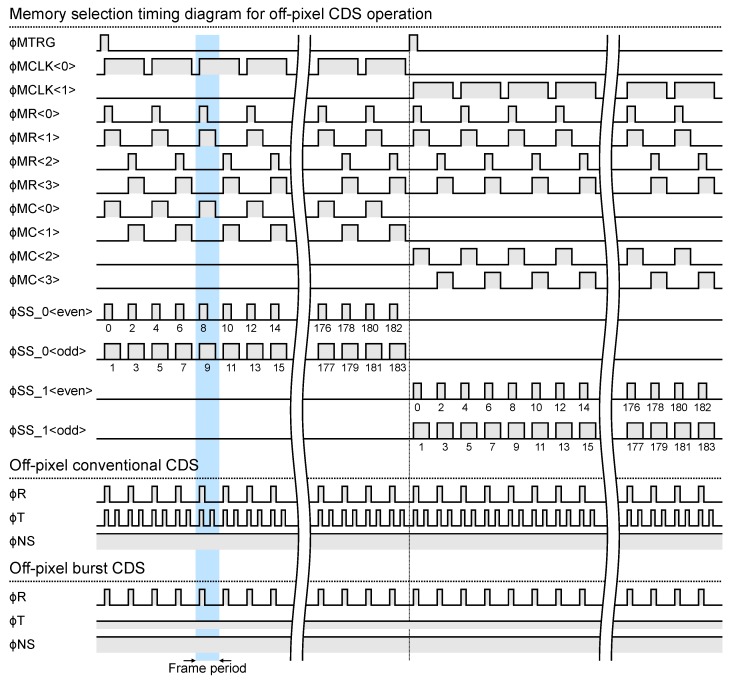
Timing diagram of the memory selection pulses and the pixel driving pulses at burst video capturing with off-pixel CDS operation.

Figure 6 and Figure 7 show the timing diagram of the memory selection pulses and the pixel driving pulses at burst video capturing with in-pixel and off-pixel CDS operation, respectively. A combination of ϕMTRG, ϕMCLK<0:1>, ϕMR<0:3> determines the memory address. During the in-pixel CDS operation, the memories are sequentially selected one by one for each frame according to the sequence shown in Figure 6. On the other hand, during the off-pixel CDS operation, two memories are selected for each frame. This is for storing the reset signal and the light signal separately during off-pixel CDS operation. After the last memory is selected, the operation returns to the selection of the first memory, and the selection of the memory is repeated until a video capturing end trigger signal is input.

**Figure 8:**
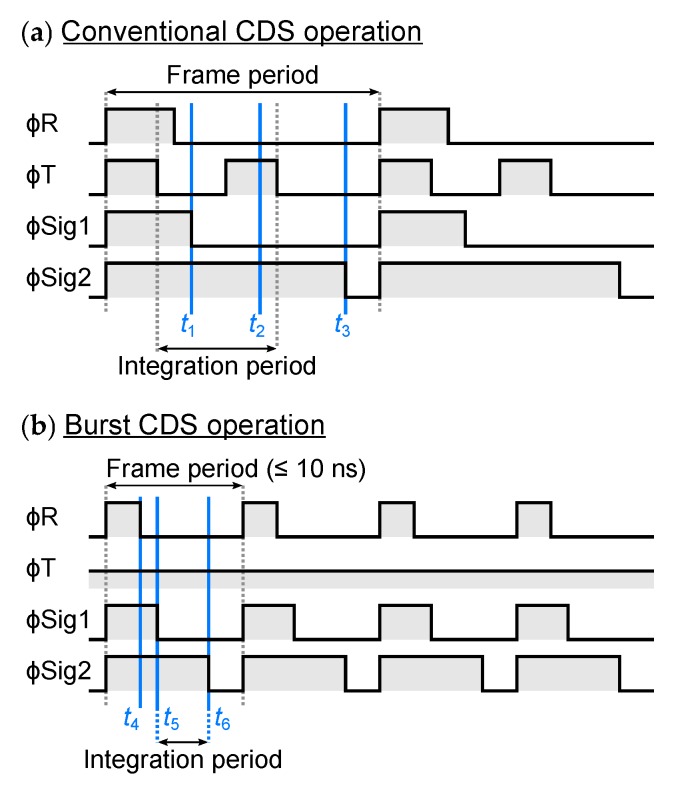
Timing diagram of pixel pulses at (**a**) conventional CDS operation with switching transfer gate, and (**b**) burst CDS operation.

Figure 8 shows the timing diagram of (a) conventional CDS operation and (b) the burst CDS operation [13], respectively. Here, ϕR is pixel reset pulse, ϕT is transfer pulse. ϕSig1 and ϕSig2 are sampling pulses of reset and signal levels, respectively. During the in-pixel CDS operation, ϕSig1 and ϕSig2 correspond to ϕNS and the memory select pulse ϕSS in Figure 4, respectively. During the off-pixel CDS operation, ϕSig1 and ϕSig2 are the memory select pulses used to select a different memory cell.

**Figure 9:**
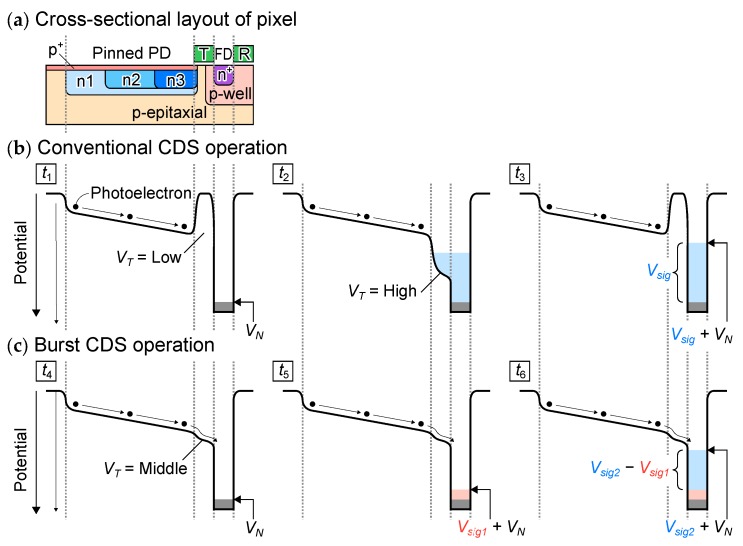
Cross sectional potential diagram of the pixel of the developed image sensor. (**a**) shows the cross-sectional layout, (**b**) shows that of conventional CDS operation, and (**c**) shows the potential diagram of the burst CDS operation.

Figure 9 shows the cross-sectional potential diagram of the pixel for electrons along PD, T, FD, and R. Figure 9a shows the cross-sectional layout. Figure 9b,c shows the potential diagram in conventional CDS operation and the burst CDS operation, respectively. They are along with the timing from $t_{1}$ to $t_{6}$ shown in Figure 8. The built-in potential gradient or electric field of about 500 V/ is formed in fully depleted PD region by dopant concentration difference as well as the control of width of buried n-type layer [16]. The photoelectron transit time of this PD has been verified to be about 5 ns by experiments [16].

During conventional CDS operation, the following operations are repeated: resetting the PD and the FD and the thermal noise $V_{N}$ remains at FD, sampling at $t_{1}$ the first voltage signal which consists of $V_{N}$, transferring the photoelectrons integrated during the integration period $t_{3}-t_{1}$ from PD to FD at $t_{2}$, sampling at $t_{3}$ the second voltage signal which consists of $V_{N}$ and the light signal $V_{sig}$ at $t_{3}$. Then, $V_{sig}$ is obtained by subtracting the first signal from the second signal. On the other hand, during the burst CDS operation, photoelectrons are always flowing toward the FD due to the electric field of PD region. After resetting the PD and FD at $t_{4}$, the thermal noise $V_{N}$ remains at FD. Shortly after $t_{4}$, the first voltage signal which consists of $V_{N}$ and the light signal due to photoelectrons arriving at FD during $t_{5}-t_{4}$ ($V_{sig1}$) is read out at $t_{5}$. Then, after the integration period: $t_{6}-t_{5}$, the second voltage signal comprised of $V_{N}$ and light signal due to photoelectrons during $t_{6}-t_{4}$ ($V_{sig2}$) is read out at $t_{6}$. By subtracting the first signal from the second signal, light signal during the integration period $t_{6}-t_{4}$ is obtained. The subtraction of the signal is carried out by the in-pixel CDS circuit in the in-pixel CDS mode and by the off-pixel manner after reading out memory signals in the off-pixel CDS mode. During the burst CDS operation, the bias voltage of the transfer gate ($V_{T}$) was set to an intermediate voltage of about 2 V to obtain high conversion gain without potential barrier from the PD and FD. The burst CDS technique achieves a frame period of 10 ns or less thanks to minimization of the transition of the pixel driving pulses. Consequently, the frame period equivalent to the photoelectrons transit time in PD is realized.

## 3. Chip Fabrication and Measurement Results

**Figure 10:**
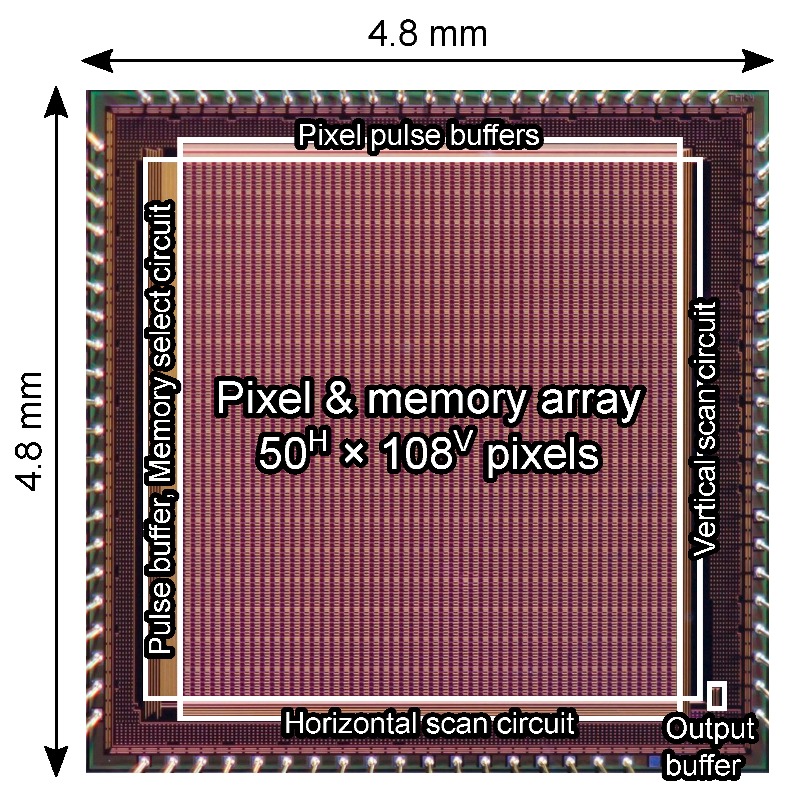
Chip micrograph of the developed burst CMOS image sensor. The number of pixels is extendable.

Figure 10 shows the fabricated chip micrograph. The chip was fabricated in LAPIS Semiconductor Miyagi 0.18 μm 1-poly-Si 5-metal-layer CMOS image sensor technology with high-density trench capacitor integration. A Si wafer with 8 μm thick p-type epitaxial layer on n-type substrate was employed by considering the optical crosstalk and the spectral sensitivity. The high-density trench capacitors were formed after the shallow trench isolation (STI) formation process. The depth of the trench capacitors is about 1.8 μm. The chip size is 4.8mm×4.8mm.

**Figure 11:**
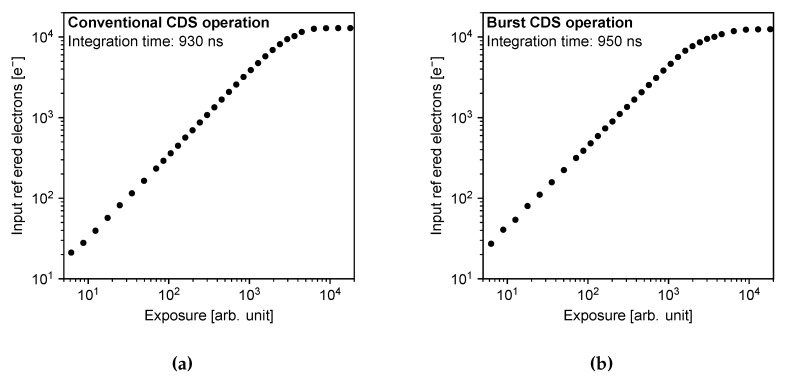
Photoelectric conversion characteristics of the developed image sensor. (**a**) shows the characteristics of conventional in-pixel CDS operation, (**b**) shows those of the in-pixel burst CDS operation. Both characteristics was taken under the frame period of 1 μs.

Figure 11 shows the photoelectric conversion characteristics of the developed sensor chip for (a) conventional CDS operation and (b) burst CDS operation. The photoelectron conversion characteristics were measured using the LB-8601 light source, manufactured by Kyoritsu Electric (Tokyo, Japan). The spectral distribution of the light source follows the standard illuminant A. The characteristics were taken under the frame period of 1 μs (equivalent to 1 Mfps frame rate) for both operation modes. The integration times were 930 ns for the conventional CDS operation and 950 ns for the burst CDS operation, respectively. The input-referred conversion gain obtained from the measurements were 104 μV/e^−^ for the conventional CDS operation and 99 μV/e^−^ for the burst CDS operation. The input-referred saturation levels in number of electrons were 13.4ke^−^ for the conventional CDS operation and 11.5ke^−^ for the burst CDS operation. The parameters were extracted from the obtained photon transfer curves. The photoelectric conversion characteristics of both operation modes show good linearity. The conversion gain of the burst CDS operation is almost the same as that of conventional CDS operation. This is because the PD is always fully depleted and only FD capacitance determines the conversion gain. The saturation of the burst CDS is slightly lower as the potential barrier toward the PD is lower compared to the conventional CDS. The fixed pattern noise and the photoresponse nonuniformity of the burst CDS operation were the same as those of conventional CDS operation. Image quality degradation due to the burst CDS operation was not confirmed.

**Figure 12:**
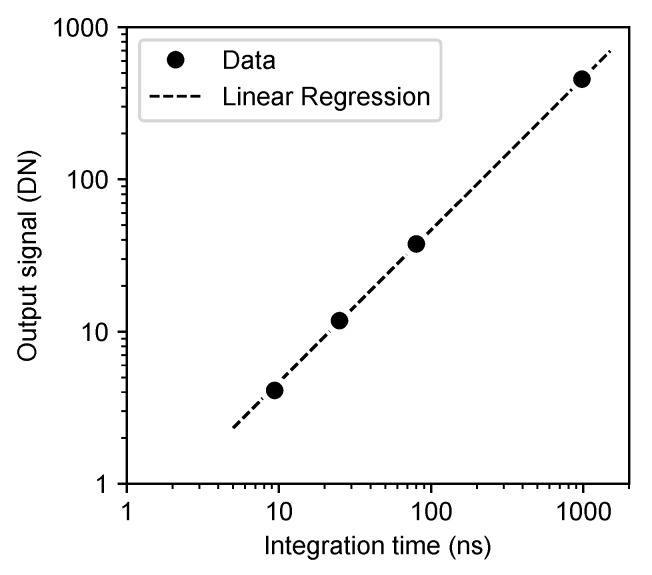
Output signal value as a function of the integration time under a constant light intensity in the in-pixel burst CDS operation.

Figure 12 shows the output signal value as a function of the integration time under a constant light intensity in the in-pixel burst CDS operation. Linearity of the output signal value to the integration time was confirmed in the integration time range from 9.4ns to 950 ns (equivalent to from 50 Mfps to 1 Mfps frame rate).

**Figure 13:**
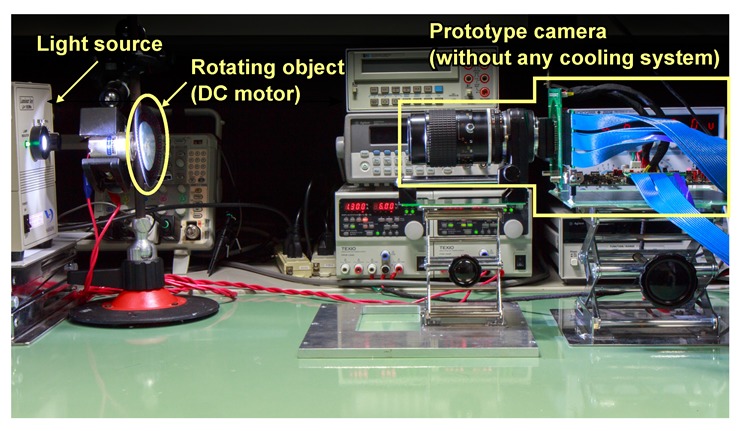
Image capturing setup. The light is irradiated from the backside of the rotating object and the transmitted light is captured by the prototype camera. The prototype camera has no cooling system.

Figure 13 shows the image capturing system for sample movie shooting. This camera was composed of a headboard mounted with the developed image sensor, an analog front end (AFE) board mounted with ADCs and voltage regulators, and a digital signal processing (DSP) board mounted with FPGA for pulse generations and signal processing. The prototype camera has no chip cooling systems.

**Figure 14:**
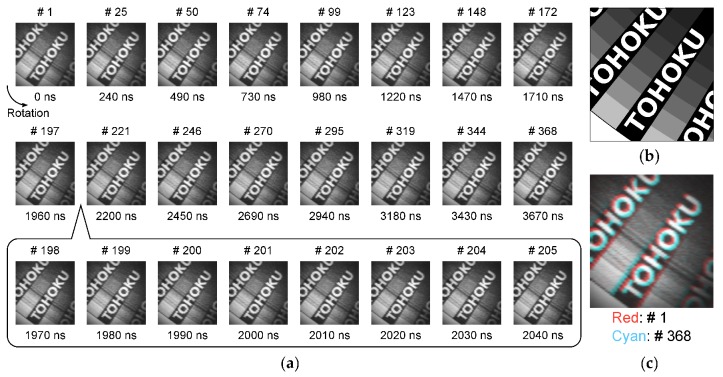
(**a**) Sample images of the rotating object (about 13 krpm) captured at 100 Mfps 368 frames with in-pixel burst CDS operation mode, (**b**) captured pattern printed on a transparent sheet, and (**c**) false-color composite image of the start and end frames, the start frame (# 1) is assigned to red channel and the end frame (# 368) is assigned to cyan channel.

Figure 14a shows the captured images of a rotating object, which is a transparent sheet with printed patterns attached to a DC motor. The light is irradiated from the backside of the object, and the transmitted light is captured. The employed light source was PFBR-600SW, manufactured by CCS Inc., (Kyoto, Japan). The rotational speed of the captured object was about 13 k rotations per minute (rpm). The captured object was a pattern printed on a transparent sheet. The pattern is shown in Figure 14b. Figure 14c shows the false-color composite image of the start and end frames of sample images, the start frame (# 1) is assigned to red channel and the end frame (# 368) is assigned to cyan channel in order to check the movement of the object during image capturing period. The color difference indicates the movement of the subject. 100 Mfps with 368 frames imaging by in-pixel burst CDS operation at room temperature was successfully confirmed with good image quality.

**Figure 15:**
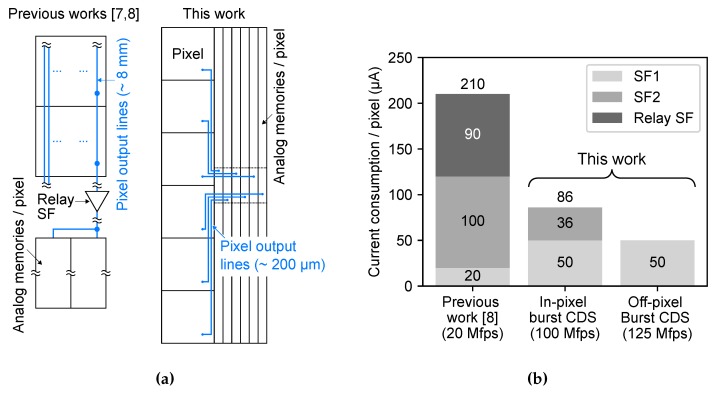
(**a**) The pixel arrangement of the previous developed burst CMOS image sensors [7,8] and this work, (**b**) Comparison of the current consumption per pixel during video capturing between the previous work [8] and this work.

The current consumption during video capturing is an important factor of the UHS imaging system. Without a large cooling mechanism, the system can be miniaturized and lightened and can be easily used in various environments. Figure 15a shows the pixel arrangement of the previously developed burst CMOS image sensors [7,8] and the developed burst CMOS image sensor of this work. In previous works, the pixel and the memory array are separated in place and connected by about 8 mm long pixel output lines with the relay source follower buffer (Relay SF). On the other hand, the pixel and the memory array in this work are placed close and connected by about 200 μm relatively short output lines. Figure 15b shows the comparison of the current consumption per pixel during video capturing at the maximum frame rate between the previous work [8] and this work. All the current values were estimated by the circuit simulation. The supply voltage of both sensors is 3.3 V. The relay SF is eliminated thanks to the pixel-wise memory array architecture. The current consumption of SF2 decreased because the pixel output load was reduced. The current consumption of SF1 increased because the maximum frame rate was increased from 20 Mfps to over 100 Mfps. As a result, the current consumption during video capturing is significantly reduced though the frame rate is increased.

**Figure 16:**
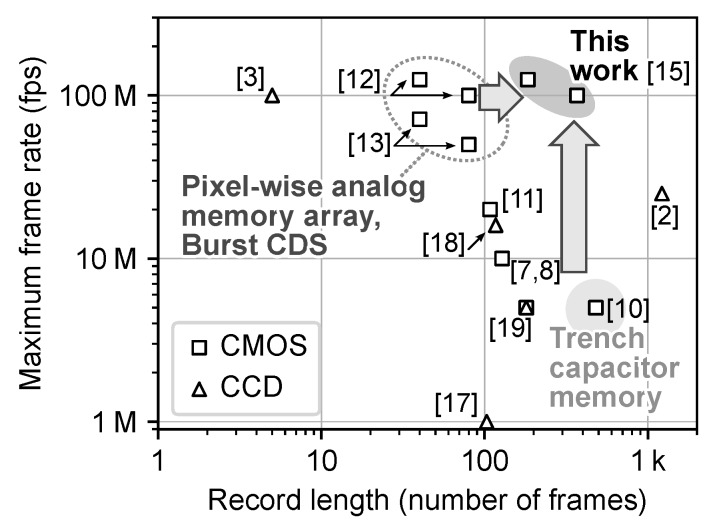
Comparison of the maximum frame rate as a function of record length for the developed chip and recently reported burst image sensors.

Figure 16 and Table 1 show the comparison of the performance for the recently reported burst image sensors [2,3,7,8,9,10,11,12,13,17,18,19]. The advanced performance of over 100 Mfps extremely high frame rate with relatively long record length of the fabricated chip was demonstrated by shortening the distance between the pixel and the memory array by the introduction of the pixel-wise analog memory array architecture and by densifying the memory by introduction of high-density trench capacitor technology.

Table 2 summarizes the performances of the developed CMOS image sensor. The maximum frame rate of the conventional CDS operation is the same as that of the previously reported burst CMOS image sensor with pixel-wise 80 analog memory array [12]. The advanced UHS video capturing performance with good photo sensing characteristics was obtained.

## 4. Conclusions

A global shutter burst CMOS image sensor with in-pixel trench capacitor memory array with over 100 Mfps and 368 record length was presented. A maximum 125 Mfps high frame rate is obtained by reduction of pixel output load by the pixel-wise memory array architecture and introduction of the burst CDS operation which minimize the pixel driving pulse transition. Long record length is realized by high density analog memory integration with Si trench capacitor. The novel performance of over 100 Mfps high frame rate video capturing with 368 record length under room temperature was confirmed. The developed CMOS image sensor technology can advance the UHS imaging technologies in scientific, engineering and medical fields.

## Figures and Tables

**Table 1:** Specification and performance comparison of reported burst image sensors.

	Sensor Type	Maximum Frame Rate (Mfps)	Record Length	Pixel Pitch (μm)	Number of Pixels	Trench Capacitor Memory	Burst CDS Operation
**This work [15]**	**CMOS**	**100**	**368**	70×35	50×108	**✓**	**✓**
Tochigi et al. [7]	CMOS	10	128	32×32	400×256		
Suzuki et al. [10]	CMOS	5	480	32×32	96×128	**✓**	
Suzuki et al. [12]	CMOS	50	80	69.12×34.56	25×100		
Suzuki et al. [13]	CMOS	100	80	69.12×34.56	25×100		**✓**
Etoh et al. [17]	CCD	1	103	66.3×66.3	312×260		
Etoh et al. [18]	CCD	16	117	43.2×43.2	362×456		
Benhammadi et al. [19]	CCD/CMOS	5	180	30×30	924×768		
Wu et al. [11]	CMOS	20	108	30×30	32×84		
Dao et al. [2]	CCD	25	1220	72.56×72.56	32×32		
Nguyen et al. [3]	CCD	100	5	12.73×12.73	512×576×2		

**Table 2:** Performance summary of the developed burst UHS CMOS image sensor.

Items	Unit	Value			
Process technology		1P5M 0.18 μm CMOS			
Power supply voltage	V	3.3			
Pixel pitch (3D stacking equivalent)	μm	$70^{H}\times 35^{V}$ $\left(35^{H}\times 35^{V}\right)$			
Photodiode size	μm^2^	$30.00^{H}\times 21.34^{V}$			
Number of pixels		$50^{H}\times 108^{V}$			
Number of analog memories/pixel		368			
		Burst CDS operation		Conventional CDS operation	
		In-pixel	Off-pixel	In-pixel	Off-pixel
Maximum frame rate	Mfps	100	125	50	71.4
Record length		368	184	368	184
Conversion gain (measured at 1 Mfps)	μV/e^−^	99		104	
Saturation (measured at 1 Mfps)	ke^−^	11.5		13.4	
